# Plasma Fibrinogen Levels and Cardiovascular Risk Factors in Japanese Schoolchildren

**DOI:** 10.2188/jea.16.64

**Published:** 2006-03-14

**Authors:** Chie Fujii, Hisataka Sakakibara, Takaaki Kondo, Hiroshi Yatsuya, Koji Tamakoshi, Hideaki Toyoshima

**Affiliations:** 1 Department of Public Health/Health Information Dynamics, Nagoya University Graduate School of Medicine.; 2 Department of Public Health and Community Nursing, Nagoya University School of Health Sciences.; 3 Department of Medical Technology, Nagoya University School of Health Sciences.

**Keywords:** Fibrinogen, Cardiovascular Diseases, Risk Factors, Child, Arteriosclerosis

## Abstract

**BACKGROUND:**

Plasma fibrinogen level has been recognized as an independent risk factor for atherosclerosis and its thrombotic complications in adults. The present study aimed to clarify the association between plasma fibrinogen levels and cardiovascular risk factors in Japanese children.

**METHODS:**

A total of 294 schoolchildren (145 boys and 149 girls) aged 10–13 years in a town in Nagano Prefecture, Japan, were surveyed in 2000 for body mass index (BMI), plasma fibrinogen, serum C-reactive protein (CRP), serum total cholesterol, serum high-density lipoprotein (HDL) cholesterol, hemoglobin (Hb) A_1c_, and ratio of serum total cholesterol to serum HDL cholesterol (TCHR).

**RESULTS:**

The mean value and standard deviation of plasma fibrinogen level among the schoolchildren was 226.0 ± 39.7 mg/dL for boys and 245.3 ± 40.9 mg/dL for girls; significantly higher for girls. Among plasma fibrinogen tertiles, serum CRP tended to increase with plasma fibrinogen in both boys and girls. An increasing trend was also found in serum total cholesterol in boys, and in TCHR, HbA_1c_ and BMI in girls. Multiple linear regression analysis revealed significant associations of plasma fibrinogen with serum CRP and HbA_1c_ in both sexes, with TCHR in boys, and with BMI in girls.

**CONCLUSIONS:**

Plasma fibrinogen levels were associated with cardiovascular risk factors such as serum CRP, TCHR, HbA_1c_, and BMI in Japanese schoolchildren.

Plasma fibrinogen levels have recently been recognized as an independent risk factor for cardiovascular diseases in adults.^1^^-^^5^ A meta-analysis suggests that the relative risk for cardiovascular diseases is 1.99 (95% confidence interval [CI]: 1.85-2.13) in persons with high plasma fibrinogen.^6^ The relative risk for coronary heart disease was reported to be 4.8 (95% CI: 1.4-16.8) in Japanese with high plasma fibrinogen.^7^ Plasma fibrinogen is a major contributing factor to blood viscosity and platelet aggregation, which play important roles in the formation of thrombi, resulting in myocardial and cerebral infarctions concomitantly with the progression of arteriosclerosis.^8^^,^^9^ Moreover, an association between atherosclerosis and inflammatory reactions has been recognized in recent years, and plasma fibrinogen levels have been studied from the viewpoint of inflammatory reactants.^10^^-^^12^

Meanwhile, from an autopsy study of subjects aged 15–19 years, McGill et al. reported that early atherosclerotic changes were seen in 20% of abdominal aortas and several percent of coronary arteries, and that these changes were related to cardiovascular risk factors including low serum high-density lipoprotein (HDL) cholesterol, hypertension, obesity, and impaired glucose tolerance.^13^ Thus, atherosclerosis appears to begin as early as in childhood, which would indicate the importance of atherosclerosis prevention measures starting at a young age.^14^^-^^17^

While plasma fibrinogen levels have been examined in adults, there are only a few studies on plasma fibrinogen in children,^18^^-^^25^ and none with respect to Japanese children. We therefore conducted a cross-sectional study to clarify the plasma fibrinogen levels in Japanese schoolchildren and the association between plasma fibrinogen and cardiovascular risk factors among them.

## METHODS

### Subjects

The subjects were a total of 330 schoolchildren aged 10–13 years in the fifth grade (elementary school) and the eighth grade (junior high school) in town M in Nagano Prefecture, who took an annual school health examination in April 2000. Almost all the schoolchildren in the fifth and eighth grades in the town participated in the health examination. They consisted of 161 fifth grade schoolchildren (82 boys and 79 girls) and 169 eighth grade schoolchildren (82 boys and 87 girls). On that occasion, we conducted additional blood examinations in a total of 294 children (145 boys, 78 fifth graders and 67 eighth graders; and 149 girls, 79 fifth graders and 70 eighth graders), whose guardians had been informed about this study with a descriptive letter and provided written consent from among a total of 330 schoolchildren (participation rate: 89.1%).

### Biochemical and Anthropometric Measurements

Blood examinations were conducted on plasma fibrinogen, serum C-reactive protein (CRP), serum total cholesterol, serum high-density lipoprotein (HDL) cholesterol, hemoglobin (Hb) A_1c_, and ratio of serum total cholesterol to serum HDL cholesterol (TCHR). Blood samples were drawn from non-fasting subjects, and taken from an antecubital vein with the subjects seated. Plasma fibrinogen and serum CRP were analyzed in a commercial laboratory (SRL, Inc. Japan), while the other items were analyzed at the Central Public Health Laboratory in Nagano Prefecture. To measure plasma fibrinogen levels, 1.8 mL of blood were put into a blood-collecting tube containing 0.2 ml of 3.8 % sodium citrate. Plasma was separated by centrifuging at 2,000 g for 10 min, and stored frozen at a temperature of less than −20°C until analysis on the next day after blood collection. Plasma fibrinogen levels were measured by thrombin clotting time, which was determined according to the method of Clauss.^26^ Serum samples were also centrifuged after blood collection and were stored in a refrigerator until analysis. Serum CRP level was measured using a latex-enhanced nephelometry method. Serum total cholesterol level was measured by enzymatic assay. Serum HDL cholesterol level was determined by a direct quantitative assay. HbA_1c_ level was measured by latex agglutination immunoassay.

Body mass index (BMI; body weight (kg) / height (m)^2^) was calculated from height and weight measured in an annual health examination in 2000.

### Statistical Analysis

Statistical analysis was done using SPSS^®^ 13.0J for Windows. A Student’s t-test was used to compare mean values of examination results between boys and girls. To examine the relation between plasma fibrinogen and each examination result, plasma fibrinogen was categorized separately for boys and girls into tertiles (boys: Tertile 1: up to 207 mg/dL, Tertile 2: 208–235 mg/dL, Tertile 3: 236 mg/dL or higher; girls: Tertile 1: up to 222 mg/dL, Tertile 2: 223–259 mg/dL, Tertile 3: 260 mg/dL or higher) and the mean values of the tertiles calculated for each examination result were investigated using one-way analysis of variance. In the analyses serum CRP was normalized by logarithmic transformation. A multiple linear regression analysis was then conducted to investigate the relation between plasma fibrinogen level and cardiovascular risk factors (age, log-transformed serum CRP, TCHR, HbA_1c_, and BMI).

## RESULTS

**Figure 1.**
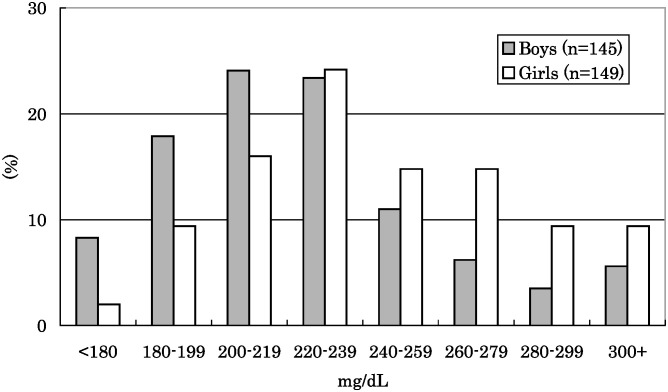
Distribution of plasma fibrinogen concentration among Japanese schoolchildren.

Mean plasma fibrinogen levels and standard deviation were 226.0 ± 39.7 mg/dL in boys and 245.3 ± 40.9 mg/dL in girls; significantly greater in girls (p<0.001) (Table 1, Figure 1). There was no significant difference in plasma fibrinogen level between fifth grade and eighth grade children in either boys or girls, although plasma fibrinogen level tended to be lower in eighth graders, particularly in boys. Serum total cholesterol level was also significantly higher in girls than in boys (p=0.036). Height was significantly higher in boys (p=0.008), while BMI was significantly greater in girls (p=0.028). No differences were seen between boys and girls in serum CRP, serum HDL cholesterol, TCHR, HbA_1c_, or body weight.

**Table 1.** Mean of examination results among Japanese schoolchildren.

	Boys				Girls				p value^‡^
	
	Total	Fifth graders	Eigth graders	p value^†^	Total	Fifth graders	Eigth graders	p value^†^	
Number of subjects	145	78	67		149	79	70		

Plasma fibrinogen (mg/dL)	226.0 (39.7)	231.7 (43.0)	219.3 (34.6)	0.059	245.3 (40.9)	246.2 (42.2)	244.3 (39.6)	0.784	<0.001
Serum C-reactive protein (mg/dL)	0.052 (0.054)	0.061 (0.069)	0.043 (0.024)	0.041	0.055 (0.054)	0.061 (0.062)	0.048 (0.042)	0.135	0.710
Serum total cholesterol (mg/dL)	168.5 (25.8)	171.2 (23.0)	165.4 (28.6)	0.180	174.9 (25.7)	174.9 (25.3)	174.9 (26.3)	0.998	0.036
Serum high-density lipoprotein (HDL) cholesterol (mg/dL)	67.5 (13.0)	68.8 (13.6)	66.0 (12.2)	0.201	67.7 (12.8)	67.9 (13.4)	67.5 (12.3)	0.847	0.869
TCHR*	2.56 (0.51)	2.56 (0.45)	2.57 (0.57)	0.876	2.64 (0.49)	2.63 (0.45)	2.65 (0.54)	0.788	0.186
Hemoglobin A_1c_ (%)	5.06 (0.31)	5.05 (0.35)	5.07 (0.27)	0.667	5.08 (0.29)	5.02 (0.31)	5.15 (0.25)	0.004	0.519
Body height (cm)	149.6 (12.6)	139.9 (5.7)	160.9 (8.2)	<0.001	146.2 (9.8)	139.0 (6.4)	154.3 (5.6)	<0.001	0.008
Body weight (kg)	40.0 (10.1)	33.0 (5.6)	48.2 (7.9)	<0.001	39.6 (10.3)	32.5 (6.6)	47.6 (7.4)	<0.001	0.720
Body mass index (kg/m^2^)	17.6 (2.1)	16.8 (2.1)	18.5 (1.8)	<0.001	18.2 (2.9)	16.7 (2.5)	19.9 (2.5)	<0.001	0.028

Standard deviations in parentheses.

*: Serum total cholesterol to serum high-density lipoprotein cholesterol ratio.

†: Difference between fifth graders and eigth graders.

‡: Difference between boys and girls in total.

The mean values for age, serum CRP, serum total cholesterol, serum HDL cholesterol, TCHR, HbA_1c_, body height, body weight and BMI are shown in Table 2 according to the plasma fibrinogen tertiles for boys and girls. As plasma fibrinogen levels increased, serum CRP level became higher in both boys and girls (trend p<0.001). A significant increasing trend was also found in serum total cholesterol in boys (trend p=0.031), and in TCHR (trend p=0.001), HbA_1c_ (trend p=0.004), and BMI (trend p=0.001) in girls. A significant decreasing trend was seen in age in boys (trend p=0.043).

**Table 2.** Mean of examination results among Japanese schoolchildren according to plasma fibrinogen concentration tertiles.

	Plasma fibrinogen (mg/dL)			p value^§^	trend p

	Tertile 1	Tertile 2	Tertile 3		
	Boys*				
	n=47	n=49	n=49		
Age (years)	11.7 (1.5)	11.3 (1.5)	11.1 (1.5)	0.125	0.043
Serum C-reactive protein (mg/dL)	0.037 (0.014)	0.035 (0.009)	0.084 (0.083)	<0.001	<0.001
Serum total cholesterol (mg/dL)	162.6 (21.3)	168.8 (22.3)	174.0 (31.6)	0.096	0.031
Serum high-density lipoprotein (HDL) cholesterol (mg/dL)	65.3 (11.5)	69.7 (13.7)	67.4 (13.5)	0.246	0.424
TCHR^‡^	2.54 (0.42)	2.51 (0.59)	2.64 (0.49)	0.415	0.335
Hemoglobin A_1c_ (%)	5.01 (0.27)	5.09 (0.32)	5.07 (0.34)	0.427	0.331
Body height (cm)	150.9 (13.5)	149.9 (12.5)	148.2 (11.9)	0.567	0.291
Body weight (kg)	41.2 (11.2)	39.8 (9.2)	39.2 (10.0)	0.628	0.344
Body mass index (kg/m^2^)	17.7 (2.3)	17.5 (1.5)	17.5 (2.4)	0.870	0.718

	Girls^†^				
	n=50	n=49	n=50		
Age (years)	11.4 (1.5)	11.5 (1.5)	11.3 (1.5)	0.775	0.843
Serum C-reactive protein (mg/dL)	0.034 (0.016)	0.038 (0.018)	0.091 (0.079)	<0.001	<0.001
Serum total cholesterol (mg/dL)	168.3 (22.9)	181.1 (26.5)	175.4 (26.4)	0.044	0.163
Serum high-density lipoprotein (HDL) cholesterol (mg/dL)	69.7 (10.7)	68.9 (15.7)	64.7 (11.2)	0.121	0.056
TCHR^‡^	2.45 (0.41)	2.70 (0.46)	2.77 (0.54)	0.002	0.001
Hemoglobin A_1c_ (%)	4.97 (0.26)	5.13 (0.32)	5.14 (0.27)	0.005	0.004
Body height (cm)	146.9 (10.1)	146.6 (9.1)	145.0 (10.1)	0.599	0.346
Body weight (kg)	38.0 (8.5)	39.5 (9.9)	41.3 (12.0)	0.269	0.106
Body mass index (kg/m^2^)	17.3 (1.9)	18.1 (2.5)	19.3 (3.7)	0.003	0.001

*: Boys : Tertile 1 : <208mg/dL, Tertile 2 : 208-235mg/dL, Tertile 3 : 236mg/dL+

†: Girls : Tertile 1 : <223mg/dL, Tertile 2 : 223-259mg/dL, Tertile 3 : 260mg/dL+

‡: Serum total cholesterol to serum high-density lipoprotein cholesterol ratio.

§: P value was calculated using one-way analysis of variance.

Multiple linear regression analyses revealed significant correlations between plasma fibrinogen and log-transformed serum CRP (boys: standardized coefficients (*β*) =0.553, p<0.001; girls: *β*=0.559, p<0.001) and HbA_1c_ (boys: *β*=0.166, p=0.015; girls: *β*=0.152, p=0.022) (Table 3). A significant correlation was also seen with TCHR in boys (*β*=0.156, p=0.024) and with BMI in girls (*β*=0.243, p=0.002). No significant correlation was seen with age in both boys and girls.

**Table 3.** Standardized regression coefficients (*β*) from the multiple linear regression analysis of plasma fibrinogen levels in relation to risk factors among Japanese schoolchildren.

	Boys (n=145)		Girls (n=149)	
	
	*β*	p value	*β*	p value
Age	-0.119	0.109	-0.113	0.154
Log-transformed serum C-reactive protein	0.553	<0.001	0.559	<0.001
TCHR*	0.156	0.024	0.073	0.265
Hemoglobin A_1c_	0.166	0.015	0.152	0.022
Body mass index	0.044	0.560	0.243	0.002

Adjusted R^2^ : 0.358 for boys, and 0.416 for girls.

*: Serum total cholesterol to serum high-density lipoprotein cholesterol ratio.

## DISCUSSION

In the present study, plasma fibrinogen levels were examined in Japanese schoolchildren aged 10–13 years. The plasma fibrinogen levels in these children were 226.0 ± 39.7 mg/dL in boys and 245.3 ± 40.9 mg/dL in girls. The plasma fibrinogen levels seem to be lower than those in Westerners, which were reportedly 245.4 mg/dL in boys and 258.8 mg/dL in girls in England and Wales among 10–11-year-old children,^18^ 353.3 mg/dL in boys and 341.8 mg/dL in girls in Spain among 10–12-year-old children,^19^ 303.0-305.0 mg/dL in boys and 302.0-307.0 mg/dL in girls in Israel among 9–14-year-old children,^20^ 303.0 mg/dL in boys and 311.0 mg/dL in girls in Germany among 4–9-year-old children,^21^ and 270.6 mg/dL in boys and 284.7 mg/dL in girls in the USA among 4–25-year-old children and youth.^22^ Plasma fibrinogen levels may be generally lower in Japanese population than Westerners, as similar findings have been reported in adults.^27^^,^^28^ The higher plasma fibrinogen level in girls than in boys shown in the present study is also consistent with earlier reports.^18^^,^^21^^,^^22^

In the present study, mean plasma fibrinogen levels tended to be lower in eighth graders aged around 13 years than in fifth graders aged around 10 years, particularly in boys, although it was not significantly different. Like the present study, a study of Spain reported that mean plasma fibrinogen tended to be lower in 13–15-year-old children than 10–12-year-old children: 353.3 mg/dL in boys and 341.8 mg/dL in girls among 10–12-year-old children; and 313.3 mg/dL in boys and 325.0 mg/dL in girls among 13–15-year-old children.^19^ On the other hand, such decreasing trend was not clearly seen in a study of Israel: 303.0 mg/dL in 9–10-year-old boys and 305.0 mg/dL in 13–14-year-old boys; and 307.0 mg/dL in 9–10-year-old girls and 302.0 mg/dL in 13–14-year-old girls.^20^ Further studies are necessary about plasma fibrinogen levels of schoolchildren in a growth period.

Plasma fibrinogen in children was significantly correlated with serum CRP, an acute inflammatory reactive protein, which was similar to the findings in adults.^12^ Both plasma fibrinogen and serum CRP are known to be hepato-synthetic acute-phase inflammatory reactants, which are regulated by interleukin-1 (IL-1), interleukin-6 (IL-6) and tumor necrosis factor alpha (TNF-*α*).^29^^,^^30^ As in adults, elevated serum CRP in children has also been shown to be related to future onset of heart disease.^31^^,^^32^ Recently an association between serum CRP concentration and intima-media thickness was reported in Finnish children.^17^ Similar to increased serum CRP concentration, the elevated plasma fibrinogen levels in children found in this study may reflect the inflammatory state of the vascular endothelium and imply early arterial changes in children.

Significant associations between plasma fibrinogen and obesity were reported for both boys and girls in previous studies,^18^^,^^21^^-^^23^ and for boys in Israel.^20^ In obese children, plasma fibrinogen levels were also associated with BMI.^24^ Obesity seems to be significantly correlated with plasma fibrinogen concentration in children as well. It was recently found that adipocytes secrete several proteins including TNF-*α* and IL-6, which can stimulate the fibrinogen synthesis in the liver in obese persons.^33^^-^^36^ Increased plasma fibrinogen synthesis rate was also observed in obese girls adolescents.^24^ In the present study a significant association between BMI and plasma fibrinogen was seen in girls but not in boys. The reason no significant association was found in boys may be that there were fewer overweight boys (6.9%) than girls (12.8%) according to a standard definition for child overweight.^37^

The present study also showed a significant association between plasma fibrinogen and TCHR in boys, though the positive association with serum total cholesterol and negative association with serum HDL cholesterol were not significant. No significant association was found between plasma fibrinogen and serum total cholesterol or serum LDL cholesterol in either a study of 9–18-year-old children in Israel,^20^ in a study of 10–11-year-old children in England and Wales,^18^ or in a study of 10–12-year-old children in Spain.^19^ A significant negative correlation with serum HDL cholesterol was found in boys in Israel,^20^ while no such relations were found in a Spanish study.^19^ Oxidized serum LDL has been shown to stimulate the release of IL-1*β*^38^ and IL-6,^39^ and to be associated with elevated serum CRP in adults.^40^ Such findings may account for the association between TCHR and plasma fibrinogen. A review by Viikari et al. indicated that lipid metabolism disorders such as high serum LDL cholesterol and low serum HDL cholesterol in children are predictors of future atherosclerosis.^41^ Attention to lipid metabolism disorders would therefore be required from childhood.^13^^,^^42^

In the present study multiple linear regression analysis revealed significant associations between HbA_1c_ and plasma fibrinogen levels in both boys and girls. Serum insulin and glucose were shown to be associated with plasma fibrinogen level in the Insulin Resistance Atherosclerosis Study (IRAS) in adults.^43^ Association was also shown between HbA_1c_ and plasma fibrinogen in Japanese adults.^12^ However, we have seen no studies on the relation between HbA_1c_ and plasma fibrinogen in healthy children. It is known that IL-6 and plasma fibrinogen concentrations are increased in patients with non-insulin-dependent diabetes mellitus in adults.^44^^,^^45^ In a study of children by Cam et al., plasma fibrinogen levels in children with insulin-dependent diabetes mellitus were significantly higher than those in non-obese healthy children.^25^ McGill et al. have reported that atherosclerosis occurs more readily in children aged 10 or more who have impaired glucose tolerance;^13^ similarly, the present findings may suggest that children with a high HbA_1c_ are more likely to have the early changes of atherosclerosis.

The present analysis of schoolchildren indicated an association between plasma fibrinogen and cardiovascular risk factors such as serum CRP, TCHR, HbA_1c_, and BMI. People with cardiovascular risk factors tended to have higher plasma fibrinogen levels even among children over the age of 10. These findings were similar to those in adults. Elevated plasma fibrinogen levels might imply early arterial changes in children. The present study was a cross-sectional study of a small number of schoolchildren in a rural area in Japan. It did not survey the secondary sex characteristics, which will need to be considered because schoolchildren are in a growth period. A longitudinal study of plasma fibrinogen levels will be further required to investigate whether elevated plasma fibrinogen of children will be a risk factor for future cardiovascular diseases.
